# Rab27a Is Essential for the Formation of Neutrophil Extracellular Traps (NETs) in Neutrophil-Like Differentiated HL60 Cells

**DOI:** 10.1371/journal.pone.0084704

**Published:** 2014-01-03

**Authors:** Tatsumi Kawakami, Jinsong He, Hiroyuki Morita, Kunio Yokoyama, Hiroaki Kaji, Chisato Tanaka, Shin-ichiro Suemori, Kaoru Tohyama, Yumi Tohyama

**Affiliations:** 1 Division of Biochemistry, Faculty of Pharmaceutical Sciences, Himeji Dokkyo University, Himeji, Japan; 2 Division of Gastroenterological Surgery, Kobe University Graduate School of Medicine, Kobe, Japan; 3 Department of Laboratory Medicine, Kawasaki Medical School, Kurashiki, Japan; Cincinnati Children's Hospital Medical Center, United States of America

## Abstract

Neutrophils play a crucial role in host defence. In response to a variety of inflammatory stimulation, they form neutrophil extracellular traps (NETs). NETs are extracellular structures composed of chromatin fibers decorated with antimicrobial proteins and developing studies indicate that NETs contribute to extracellular microbial killing. While the intracellular signaling pathways that regulate NET formation remain largely unknown, there is growing evidence that generation of reactive oxygen species (ROS) is a key event for NET formation. The Rab family small GTPase Rab27a is an important component of the secretory machinery of azurophilic granules in neutrophils. However, the precise mechanism of NET formation and whether or not Rab27a contributes to this process are unknown. Using neutrophil-like differentiated HL60 cells, we show here that Rab27a plays an essential role in both phorbol myristate acetate (PMA)- and *Candida albicans*-induced NET formation by regulating ROS production. Rab27a-knockdown inhibited ROS-positive phagosome formation during complement-mediated phagocytosis. To investigate the role of Rab27a in neutrophil function in detail, both primary human neutrophils and neutrophil-like differentiated HL60 cells were treated with PMA, and NET formation process was assessed by measurement of release of histone H3 into the medium, citrullination of the arginine in position 3 of histone H4 and chase of the nuclear change of the living cells in the co-existence of both cell-permeable and -impermeable nuclear indicators. PMA-induced NET formation occured sequentially in both neutrophil-like differentiated HL60 cells and primary neutrophils, and Rab27a-knockdown clearly inhibited NET formation in association with reduced ROS production. We also found that serum-treated *Candida albicans* triggers NET formation in a ROS-dependent manner, and that Rab27a-knockdown inhibits this process as well. Our findings demonstrate that Rab27a plays an important role in NET formation induced by both *Candida albicans* infection and PMA treatment by regulating ROS production.

## Introduction

Rab27a is a member of the Rab family of small GTPase proteins. The Rab GTPases control almost all membrane trafficking processes, including vesicle budding, docking and fusion to acceptor membranes, and exosome release [1], [2]. Rab27a is involved in the exocytosis of secretory granules in melanocytes and cytotoxic T lymphocytes. Mutations in Rab27a cause type-2 Griscelli syndrome, which is characterized by pigment dilution and defects in cytotoxic granule transport, and aslo cause macrophage activation syndrome (known as hemophagocytic syndrome, HS) [3]–[5]. Rab27a also plays a critical role in innate immune defenses against invading microorganisms. We previously demonstrated that Rab27a negatively regulates complement-mediated phagocytic activity in association with F-actin remodeling in macrophages [6]. In addition, Rab27a-dependent recruitment of NADPH oxidase reportedly prevents acidification of phagosomes and limits proteolytic activity for antigen cross-presentation in dendritic cells [7]. Catz and colleagues intensively studied the important role of Rab27a in myeloperoxidase (MPO) secretion in neutrophil azurophilic granules [8].

Neutrophils are the most abundant white blood cells in the peripheral blood, and play a crucial role in the innate immune system. Neutrophils use several strategies to eliminate microorganisms, such as phagocytosis, generation of reactive oxygen species (ROS), and secretion of granular antimicrobial molecules. In addition to these mechanisms, another pathogen-killing machinery has been identified, termed neutrophil extracelluar traps (NETs) [9]. NETs are extracellular structures composed of chromatin fibers decorated with antibacterial peptides or enzymes, and they appear to play an important role in host defense against microorganisms [9]–[11]. It was recently reported that impaired clearance of NETs is involved in the development of autoimmune diseases, such as anti-neutrophil cytoplasmic antibody-associated vasculitis, and systemic lupus erythematosus (SLE) [12]–[14]. Although recognition of the importance of NETs in immunobiology is growing, the factors that determine or facilitate the formation of NETs remain unknown. An essential role for ROS in NET formation has been shown pharmacologically, and, more relevantly, from data indicating that neutrophils from patients with mutations in any of the subunits of the NADPH oxidase enzyme complex cannot produce ROS nor form NETs [15]–[17]. Zychlinsky and colleagues reported that MPO, an enzyme that acts downstream of NADPH oxidase, is required for NET formation, and that both MPO and neutrophil elastase stored in azurophilic granules translocate to the nucleus upon activation and ROS production [18]. These authors also showed that neutrophils from completely MPO-deficient donors do not form NETs after phorbol myristate acetate (PMA) treatment, while partially MPO-deficient neutrophils can form NETs [19].

In this study, we investigated the role of Rab27a in neutrophil function using primary human neutrophils and neutrophil-like differentiated HL60 cells and found that this protein is essential for both PMA- and *Candida albicans* (*C. albicans*)-induced NET formation by up-regulating ROS producton mediated by NADPH oxidase.

## Materials and Methods

### Ethics Statement

This work handled human neutrophils from peripheral blood and was performed under the approval by the Institutional Research Ethics Committee of Himeji Dokkyo University (approval number 13-01). All participants provided written informed consent for this study. Consent forms were approved for use by the above Ethics Committee.

### Isolation of Human Neutrophils

Primary human neutrophils were isolated from acid-citrate dextrose anticoagulated venous blood of healthy volunteers with Ficoll-Hypaque Polymorphprep™ solution (GE Healthcare Bio-Sciences AB Uppsala Sweden) under the approval by Himeji Dokkyo University Institutional Review Board.

### Cell Preparation

A human leukemia cell line HL60 was maintained in RPMI 1640 medium, supplemented with 8% heat-inactivated fetal calf serum (FCS), 100 U/ml penicillin and 100 µg/ml streptomycin in 5% CO_2_ humidified air at 37°C. The cells were induced to differentiation into neutrophil-like cells by treatment with 1 µM all-trans retinoic acid (ATRA) (Sigma) for 3 days as previouly described [20]. Cell differentiation was confirmed morphologically by May-Gruenwald-Giemsa staining of the cytospin preparations and by evaluating complement receptor3 (CR3) expression with flow cytometry.

### Antibodies and Reagents

Rabbit anti-human Rab27a polyclonal antibody (polyAb), mouse anti-α tubulin monoclonal antibody (mAb) was purchased from Sigma (St. Louis, MO). Mouse anti-human CD11b (complement receptor 3, CR3) mAb for flow cytometry was purchased from DAKO (Glostrup, Denmark). Rabbit anti-human MPO polyAb, rabbit anti-human histone H3 mAb (D1H2) and mouse anti-human histone H4 mAb (L64C1) was from Cell Signaling Technology (Danvers, MA). Rabbit anti-human histone H4 (citrulline 3) polyAb was from Millipore (Billerica, MA). Texas Red-conjugated zymosanA, 5-(and-6)-chloromethyl-2′,7′-dichlorodihydrofluorescein diacetate, acetyl ester (CM-H_2_DCFDA) and Sytox Green were purchased from Lifetechnologies (Carlsbad, CA). Puromycin, phorbol 12-myristate 13-acetate(PMA) and diphenylene iodonium chloride (DPI), an NADPH oxidase inhibitor were purchased from Sigma (St. Louis, MO) and RPMI1640 medium was from Wako (Osaka, Japan). Penicillin-streptomycin mixed solution was from Nacalai Tesque (Kyoto, Japan), and polybrene was purchased from Millipore (Bedford, MA). Aminophenyl fluorescein (APF) and hydroxyphenyl fluorescein (HPF) were purchased from Sekisui Medical (Tokyo, Japan). Hoechst33342 was purchased from Dojindo Laboratories (Kumamoto, Japan). DNaseI was purchased from Wako (Osaka, Japan).

### Plasmids and Transfection

To inhibit *Rab27a* gene expression, a vector for short hairpin RNA (shRNA) incorporated in pLKO.1-puro (Sigma-Aldrich, Mission shRNA code: TRCN 0000005294) and a vector for shRNA control (Sigma-Aldrich, Non-Target shRNA) were transfected into HL60 cells by lentiviral system and positive clones were selected with 1 µg/ml puromycin.

### Phagocytosis Assay

Complement-mediated phagocytosis assay was performed as previously described [6], [21]. Briefly, to opsonize zymosan particles with C3bi, complement activation cascade in serum was utilized. Texas Red-conjugated zymosan A particles were incubated in 50% human serum at 37°C for 30 min and then washed with PBS twice at 4°C. C3bi-opsonized or non-opsonized zymosan particles were added to neutrophil-like differentiated HL60 cells or mutant cells (ratio of cell: zymosan particle, 1∶10) and incubated for indicated times in the presence or absence of fluorescent ROS indicator (APF or HPF) at 37°C and analyzed by flow cytometry (FACS Calibur, Becton Dickinson). To analyze the phagosome maturation process, phagocytosis assay was performed using live cell imaging sysytem of confocal fluorescent microscopy with an LSM 510 laser scanning unit (Carl Zeiss, Oberkochen, Germany).

### Determination of ROS Production by Flow Cytometry

Cells at a density of 2×10^5^ cells/ml were treated with 100 ng/ml PMA and then incubated for further 30 min at 37°C in the presence of CM-H_2_DCFDA (1 µM) or for further 20 min in the presence of APF (2 µM), and subjected to flow cytometry.

### Immunoblotting Analysis

Cells were lysed with lysis buffer (1% Triton X-100, 50 mM Tris-HCl (pH7.2), 100 mM NaCl, 5 mM EDTA, 1 mM phenylmethylsulfonyl fluoride (PMSF)) and lysate was mixed with 3× Laemmli sample buffer. Proteins were separated by SDS-PAGE and transferred to a polyvinylidene difluoride membrane (Millipore). The membrane was blocked with 5% skim milk in T-TBS (25 mM Tris-HCl (pH 8.0), 150 mM NaCl, 0.1% Tween 20) for 60 min at room temperature and then incubated with the appropriate antibodies. The membrane was washed three times with T-TBS and incubated with horseradish peroxidase-conjugated goat anti-rabbit or anti-mouse antibodies for 30 min, and specific proteins were detected using an enhanced chemiluminescence immunoblotting system and a lumino-image analyzer (LAS-3000, Fuji Photo Film, Tokyo, Japan).

### PMA-induced NET Formation

Primary human neutrophils or neutrophil-like differentiated HL60 cells and mutant cells were allowed to adhere to 12-mm in diameter round glass coverslips and then stimulated with 100 ng/ml PMA for indicated times and then fixed with 4% paraformaldehyde (PFA) in PBS for immunofluorescence staining or with 2.5% glutaraldehyde in 0.1 M pH 7.4 phosphate buffer for scanning electron microscopy. For immunofluorescence staining, the samples were permealized with 0.5% TritonX100 in PBS for 1 min, blocked wirh 5% BSA in PBS for 30 min. Next, the corresponding antibody in PBS containing 0.05% BSA was applied for 1 hour at room temperature. After washing with PBS, AlexaFluor 488-conjugated secondly antibody solution containing Hoechst 33342 was applied for 45 min at room temperature. After washing, NETs were analyzed using LSM 510 confocal lazer-scanning microscope (Carl Zeiss).

For scanning electron microscopy, the glass coverslips containing the fixed cells were transferred into the culture plate containing OsO_4_ for 30 min, washed with water, transferred into the plate containing1% tannic acid for 30 min, and transferred again into the plate containing OsO_4_ for 30 min after washing with water. Then, dehydration was performed by wasing with gradual high-concentrations of ethanol. The samples were dried with critical point dryer and the surface was coated with 5 nm platin/carbon layer using a thin layer evaporator, and observed using S-3400N scanning electron microscope (Hitachi High-Tech Corp., Tokyo, Japan ).

For evaluation of NET formation, the total histone H3 content in the culture supernatant was assesed as follows. After the stimulation with PMA for indicated times, primary neutrophils or neutrophil-like differentiated HL60 cells were incubated with new media containing DNaseI (40 U/mL) for 15 min at room temperature to promote the break down of the nucleus and release of NETs formed in response to stimulation. The supernatant was gently removed and centrifuged at 420 *g* for 5 min. The cell-free supernatant was mixed with 3× Laemmli sample buffer prior to immunoblotting analysis. Citrullination of the arginine in position 3 of histone H4 was assesed by immunoblotting using spsecific antibody against histone H4 citrullinated on residue3 (H4cit3). For live cell imaging, the living cells were treated with a mixture of cell permeable (Hoechst 33342) and cell-impermeable (Sytox Green) DNA fluorescent dyes. The change of cell shape was monitored using live cell imaging system (CO_2_ conc 5%, at 37°C ) of confocal microscopy with an LSM 510 laser scanning unit during 4 h. Cell stages up to NET formation was classified into 4 groups by the degree of nuclei expansion and types of staining patterns.

### *C. albicans*-induced NET Formation

*C. albicans* was routinely cultured in Sabouraud dextrose broth for 18 h until the stationary phase, at 30°C under shaking and sub-cultured on Sabouraud dextrose agar for 24 h. After sub-culture, the spore number was counted and incubated in 50% human serum at 37°C for 30 min and then washed with PBS twice at 4°C. Serum-treated or non-treated *C. albicans* were added on neutrophil-like differentiated HL60 cells or mutant HL60 cells (the ratio of *C. albicans* to cell; 5∶1) and incubated for 3 hours at 37°C in the presence of both Hoechst 33342 and Sytox Green and monitored using live cell imaging system as described in the case of NET formation using PMA. Cell stages up to NET formation was classified into 3 groups by types of staining reagents and the degree of nuclei expansion; nuclei are stained with Hoechst 33342 (corresponding to stage1 or 2 in PMA-activation); chromatin is stained with Sytox Green with spherical form (corresponding to stage3 in PMA-activation); chromatin stained with Sytox Green is further decondensed in a cloud-like spread form (corresponding to stage4 in PMA-activation). In some cases, cells were pretreated with 1 µM DPI.

### Statistical Analysis

In some experiments, statistical significance was determined by the Student’s *t*-test.

## Results

### Neutrophil-like Differentiation of Rab27a-knockdown HL60 Cells

**Figure 1:**
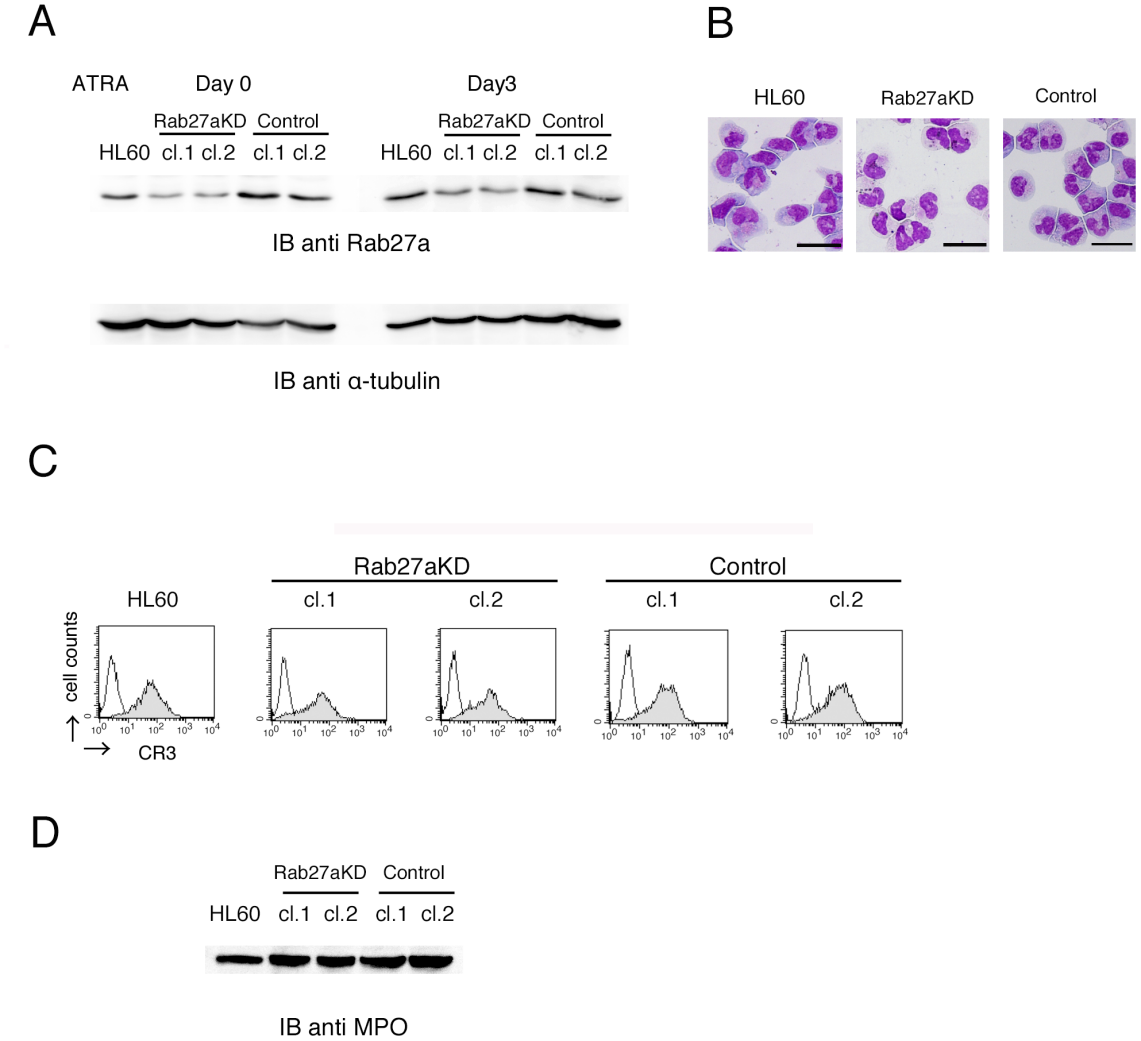
Effects of Rab27a-knockdown on neutrophil-like differentiaiton of HL60 cells. (A–C) HL60 cells, Rab27a-knockdown cells transfected with shRNA-Rab27a using a lentiviral system (Rab27aKD; clone 1 and clone 2) and control-shRNA transferred HL60 cells (Control; clone 1 and clone 2) were treated with ATRA for 3 days. (A) The expression of Rab27a in similarly treated cells were analyzed by immunoblotting analysis. (B) Morphology and (C) cell surface expression of CR3 were analyzed by May-Giemsa staining and flow cytometry, respectively. In (B), Rab27aKD clone 1 and Control clone 1 are shown as representative images. (D) Expression of myeloperoxidase (MPO) in whole cell lysates were analyzed by immunoblotting analysis. Scale bars indicate 10 µm.

To elucidate the role of Rab27a in neutrophil function, HL60 cells, Rab27a-knockdown cells transfected with shRNA-Rab27a using a lentiviral system, and control-shRNA transferred HL60 cells were treated with all-trans retinoic acid (ATRA) and allowed to differentiate into neutrophil-like cells for 3 days [6], [20]. Reduced expression of Rab27a in Rab27a-knockdown cell clones after differentiation was confirmed by immunoblotting analysis (Fig. 1A). Nuclear lobulation (Fig. 1B), cell surface expression of complement receptor 3 (CR3) (Fig. 1C), and expression of MPO in whole cell lysates were not affected by Rab27a-knockdown (Fig. 1D).

### Rab27a-knockdown Suppresses Phagosome Maturation by Reducing ROS Production

**Figure 2:**
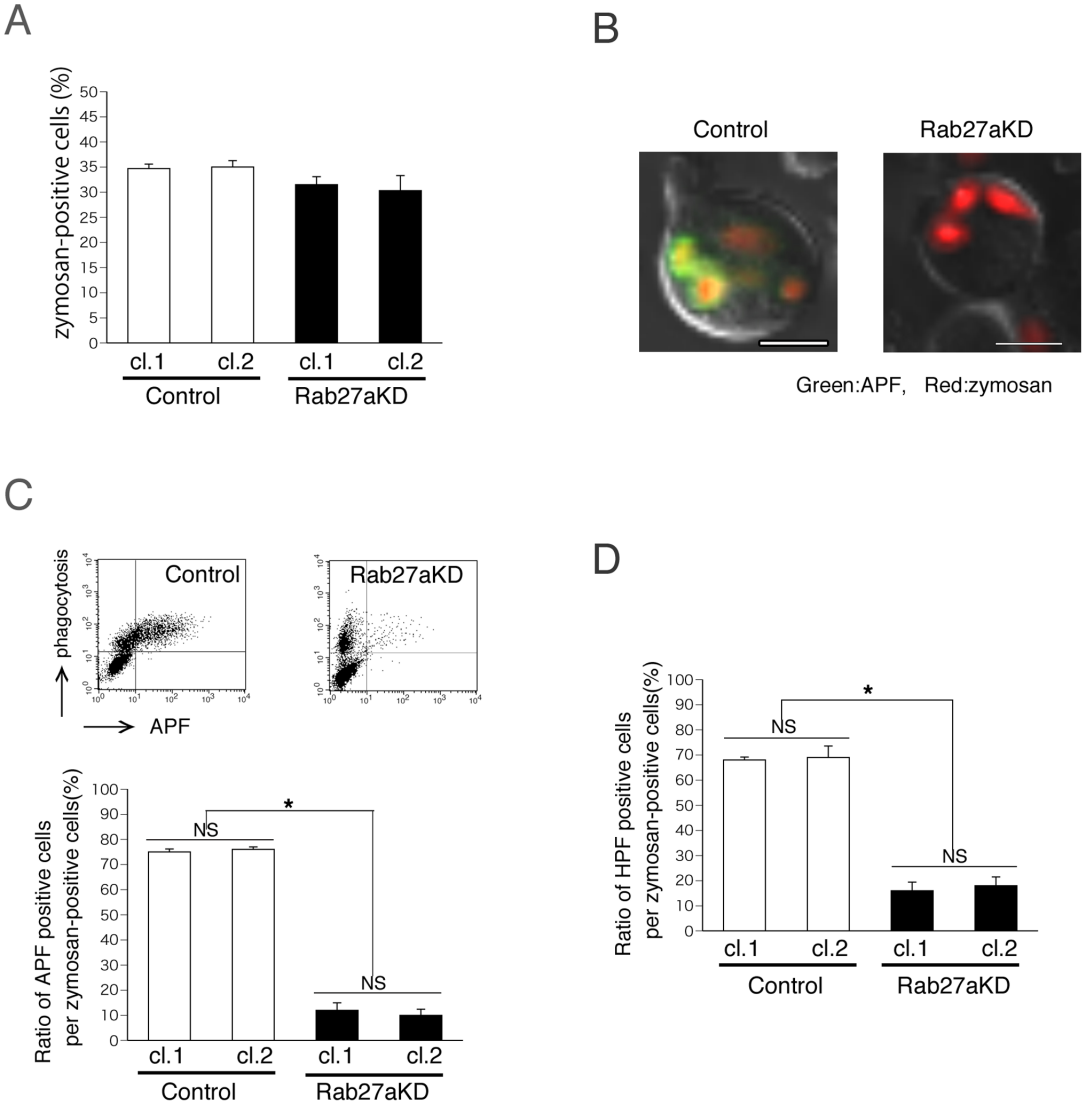
Decreased production of highly reactive oxygen species production induced by complement-mediated phagocytosis in Rab27a-knockdown cells. (A–D) Both Rab27a-knockdown HL60 cells (Rab27aKD; clone 1 and clone 2) and control-shRNA transferred HL60 cells (Control; clone 1 and clone 2) were treated with ATRA for 3 days and complement-mediated phagocytosis assay was performed using serum-opsonized Texas Red-labeled zymosan. (A) At 30 min after the onset of phagocytosis, uptake of zymosan particles in Rab27a-knockdown cells was analyzed by flow cytometry. In the presence of APF, (B) microscopic analysis and (C) flow cytometric analysis were performed at 30 min after the onset of phagocytosis. In (B) and the cytograms of (C), the results of Rab27aKD clone 1 and Control clone 1 are shown as representative data. In (B), hypochlorite appears green and Texas Red-labeled zymosan is red. Scale bars indicate 5 µm. (D) In the presence of HPF, flow cytometric analysis was performed at 30 min after the onset of phagocytosis. In (A), (C), (D), the data are the mean with SD from three independent experiments. Asterisks (*) mean that the difference is statistically significant (p values<0.01). NS means no statistical significance.

We first examined the effect of Rab27a-knockdown on complement-mediated phagocytosis in neutrophil-like differentiated cells, using serum-opsonized fluorescence-labeled zymosan, as previously described [6], [21]. At 30 min after the onset of phagocytosis, uptake of zymosan particles by Rab27a-knockdown cells was almost equal to that of control-shRNA cells (Fig. 2A). This result suggested that Rab27a is not involved in the initial step of complement-mediated phagocytosis, C3bi-mediated recognition and internalization by neutrophil-like differentiated HL60 cells, in contrast to macrophage-like differentiated cells, as we previously reported (6). Next, we examined MPO activity of phagosomes using aminophenyl fluorescein (APF), a selective fluorescent indicator for hypochlorite, the primary product of MPO [22], [23]. Microscopic analysis showed that 15 to 30 min after the onset of phagocytosis, hypochlorite-positive phagosomes gradually appeared and increased in number in control shRNA cells, but this phenomenon was much less pronounced in Rab27a-knockdown cells (Fig. 2B).

Flow cytometric analysis was performed to quantitatively assess the effect of Rab27a-knockdown on phagocytosis-induced MPO activity. As shown in Figure 2C, the ratio of APF (hypochlorite)-positive cells to zymosan particle-positive cells was significantly reduced as a result of Rab27a-knockdown. These results suggest that Rab27a plays an essential role in the process of MPO activation induced by complement-mediated phagocytosis.

Hydroxyphenyl fluorescein (HPF), a fluorescent indicator specific for highly reactive oxygen species other than hypochlorite, was also used to examine whether Rab27a is involved in other ROS production system besides MPO. As was the case with APF, the ratio of HPF-positive cells to zymosan particle-positive cells was significantly reduced as a result of Rab27a-knockdown from both flow cytometric and microscopic analyses (Fig. 2D and data not shown), indicating that in neutrophil-like differentiated HL60 cells, Rab27a plays a pivotal role in the production of highly reactive oxygen species induced by complement-mediated phagocytosis.

### PMA-induced NET Formation Occurs Sequentially in Neutrophil-like Differentiated HL60 Cells

Based upon our finding that Rab27a plays an essential role in regulating ROS production induced by complement-mediated phagocytosis, we hypothesized that Rab27a is also involved in NET formation.

**Figure 3:**
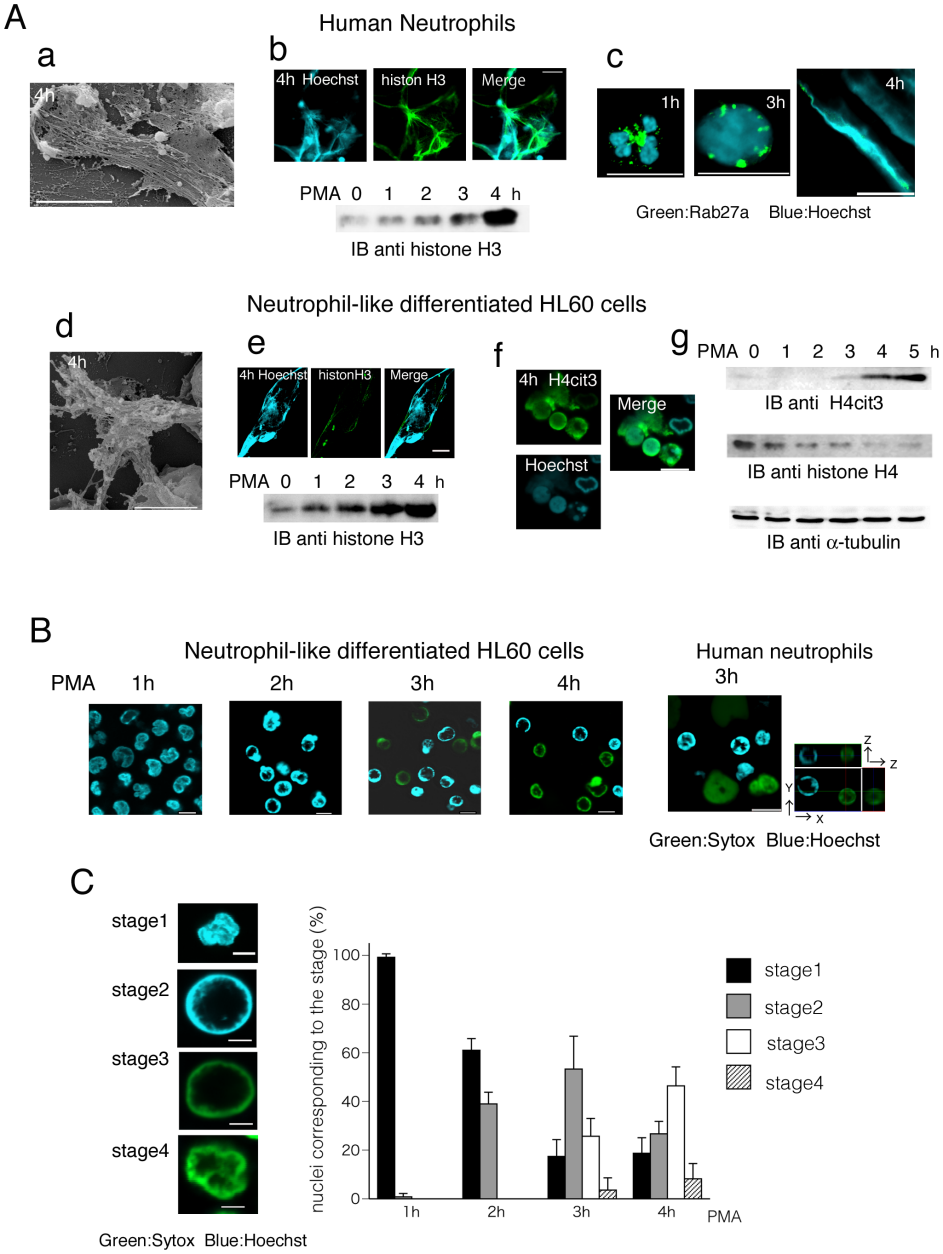
Process of NET formation after PMA treatment both in primary neutrophils and neutrophil-like differentiated HL60 cells. (A–C) Primary human neutrophils and neutrophil-like differentiated HL60 cells were stimulated with PMA. (A) NET formation was observed both in human neutrophils (a–c) and neutrophil-like differentiated HL60 cells (d–g). (a and d) Scanning electron microscopy (SEM) images of both cells indicating NET structures. Scale bars indicate 10 µm. (b and e) PFA-fixed cells were stained with antibody against histone H3 in the presence of Hoechst 33342. Scale bars indicate 20 µm. Extracellular histone H3 content in DNase I-treated supernatants were assessed by immunoblotting analysis in the time course study after PMA treatment. (c) PFA-fixed cells were stained with antibody against Rab27a in the presence of Hoechst 33342. (f) PFA-fixed cells were stained with antibody against H4cit3 in the presence of Hoechst 33342. Scale bars indicate 10 µm. (g) The amounts of histone H4cit3 and histone H4 in the cells in the time course study after PMA treatment were assesed by immunoblotting analysis using specific antibodies.(B) Both primary neutrophils and neutrophil-like differentiated HL60 cells were stimulated with PMA with a mixture of Hoechst 33342 and Sytox Green. Representative fluorescence images at indicated time points are shown. In the images of human neutrophils (right), x-y-z section images are also shown to indicate the spherical forms of the nuclei. Scale bars indicate 10 µm. Blue and green colors show Hoechst 33342-positive and Sytox Green-positive, respectively. (C) Cell stages in NET formation were classified into four groups by the degree of nuclei expansion and types of reagent staining (stage1–4). Nuclei are stained with Hoechst 33342 and showed lobulated shape and decondensation does not occur judging from z-section scanning (stage1), nuclei are stained with Hoechst 33342 and chromatin decondensation occurs like a spherical form (stage2), chromatin stained with Sytox Green like a spherical form (stage3), chromatin stained with Sytox Green shows a cloud-like spread form (stage4). The histogram shows the percentage of the cells in the individual stage at indicated time points. The data are the mean with SD from three independent experiments. Scale bars indicate 5 µm.

To define NET formation in vitro, both primary human neutrophils and neutrophil-like differentiated HL60 cells were treated with PMA, a chemical inducer of NET formation. During 4 h after PMA treatment, human neutrophils produced typical NET structures composed of chromatin (Fig. 3A-a, 3A-b upper), and the amount of histone H3 released into culture medium was gradually increased (Fig. 3A-b lower) as previouly reported [24]. Immunofluorescence staining showed that Rab27a was present in cytoplasmic granular structure before PMA treatment, Rab27a-containing granules appeared to be recruited to the decondensed chromatin within 3 h after PMA treatment, and, localization of Rab27a became faint after chromatin was released (NET formation), suggesting that Rab27a is involved in the early step of NET formation (Fig. 3A-c).

In neutrophil-like differentiated HL60 cells, similar NET structures and release of histone H3 were observed (Fig. 3A-d, 3A-e). In addition, citrullination of the arginine in position 3 of histone H4 which is catalyzed by peptidylarginine deiminase 4, was analyzed as a sign of chromatin decondensation [25]–[27]. Microscopic observation using a specific antibody against histone H4 citrullinated on residue3 (H4cit3) showed that H4cit3 appeared and colocalized with spread chromatin-staining, indicating NET formation in accordance with the previous reports (Fig. 3A-f) [27]. By immunoblotting analysis its citrullination was detected faintly at 3 h and clearly at 4–5 h after PMA treatment (Fig. 3A-g).

Furthermore, to detect NET formation process in live cell imaging, primary human neurtophils and neutrophil-like differentiated HL60 cells were treated with PMA in the presence of a mixture of cell-permeable and -impermeable nuclear indicators Hoechst 33342 and Sytox Green, respectively [24]. As shown in Figure 3B, immediately after PMA treatment, cells attached to the culture plates, the lobulated nuclei stained with Hoechst 33342 and chromatin decondensation occured like a spherical form judging from z-section scanning (2 h). A part of the decondensed chromatin turned from blue to green color, which indicated Sytox Green fluorescence 3 h after the treatment, and some of the Sytox Green-stained chromatin became diffuse and spread like a cloud showing NET-like structures. The cells were classified into the nuclear stages 1 to 4 by the staining patterns of nuclear indicators in addition to the degree of decondensation in the course of PMA treatment and the percentage of the cells in each individual stage was shown in Figure 3C. PMA-induced nuclear change occured sequentially and most of the cells lost lobulated nuclei 3 h after PMA treatment (stage 1 was less than 20%; the histogram in Figure 3C). Four hours after the treatment, over a half of the cells were stained positively with cell-impermeable nuclear indicators and about 10% of them revealed cloud-like spread form, NET-like structures (stage 4).

### Rab27a-knockdown Suppresses PMA-induced NET Formation in Association with Reduced ROS Production

**Figure 4:**
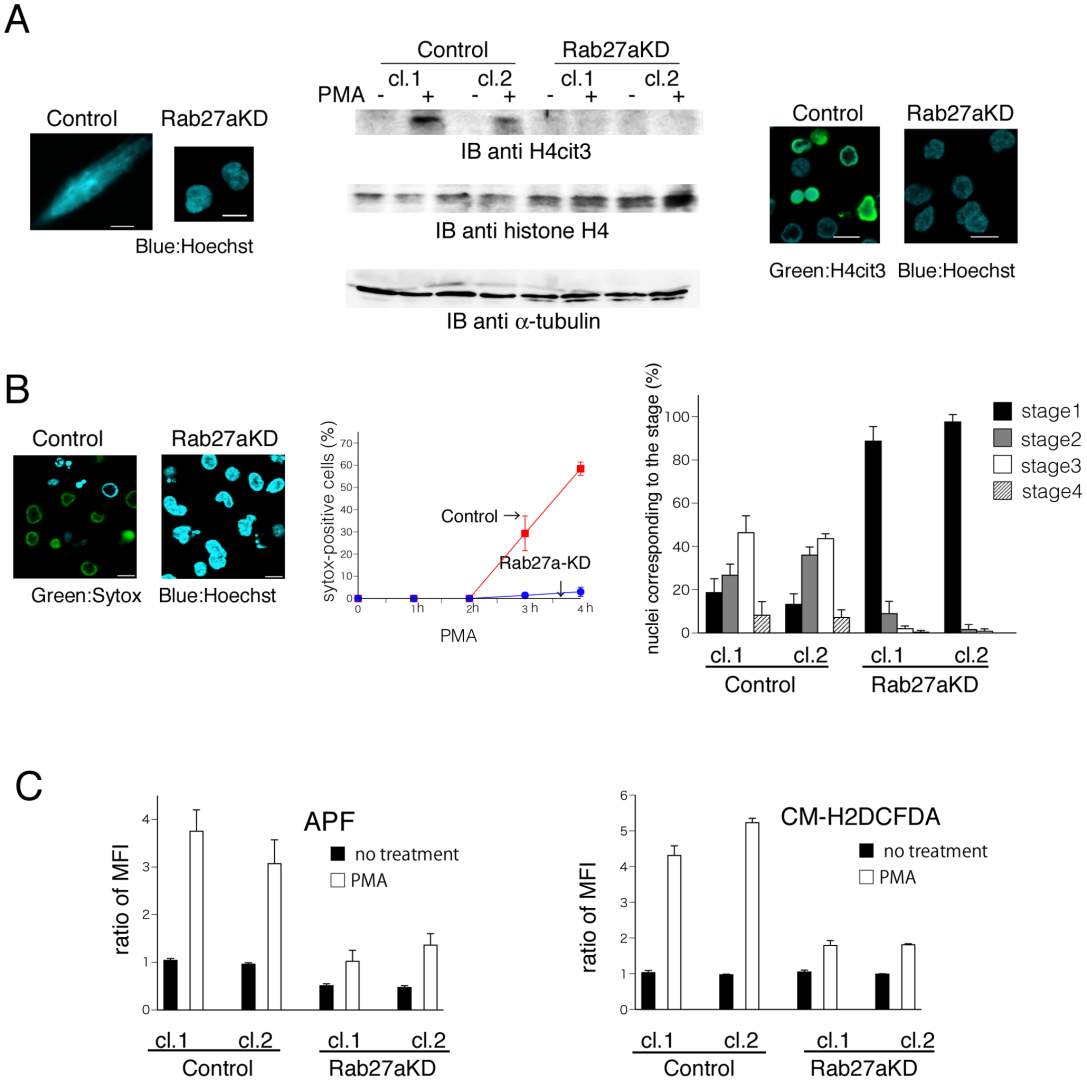
The effects of Rab27a-knockdown on PMA-induced NET formation. (A–C) Neutrophil-like differentiated control HL60 cells (Control; clone 1 and clone 2) and Rab27a-knockdown cells (Rab27aKD; clone 1 and clone 2) were stimulated with PMA. (A) Both cells fixed with PFA were stained with Hoechst 33342 (left). Cell lysates were assesed by immunoblotting analysis using specific antibody against H4cit3 and histone H4 (middle). PFA-fixed cells were stained with antibody against H4cit3 in the presence of Hoechst 33342 (right). Scale bars indicate 10 µm. (B) Representative fluorescence images at 4 hours after PMA treatment in the presence of both Hoechst 33342 and Sytox Green. Scale bars indicate 10 µm. Blue and green colors show Hoechst 33342-positive and Sytox Green-positive, respectively (left). The results of Rab27aKD clone 1 and Control clone 1 are shown as representative data. Frequency of Sytox Green-positive cells at indicated time points after PMA treatment (middle). The percentage of the cells in the individual stages at 4 h after PMA treatment (right). (C) The mean fluorescence intensity (MFI) after PMA treatment in the presence of APF at 30 min (left) or CM-H_2_DCFDA at 20min (right). The mean value of MFI of control cells was adjusted to 1. In (B) and (C), the data are the mean with SD from three independent experiments.

To determine whether Rab27a contributes to PMA-induced NET formation, the effect of Rab27a-knockdown on the production of NETs was analyzed by using neutrophil-like differentiated HL60 cells. In Rab27a-knockdown cells, NET-like structures were hardly found within 4 h after PMA treatment but a part of the control cells produced typical NET-like structures (Fig. 4A, left). Next, the effect of Rab27a-knockdown on citrullination of the arginine in position 3 of histone H4 was analyzed by using neutrophil-like differentiated cells. In Rab27a-knockdown cells, PMA-dependent appearance of H4cit3 was clearly suppressed as compared with control cells both in immunoblotting analysis (Fig. 4A, middle) and microscopic observation stained with specific antibody against H4cit3 (Fig. 4A, right). Next, the effect of Rab27a-knockdown on PMA-induced NET formation process was analyzed with a mixture of cell-permeable and -impermeable nuclear indicators In control cells, the proportion of Sytox Green-positive cells increased gradually (29% at 3 h and up to 58% at 4 h after PMA treatment). However, few Sytox Green-positive Rab27a-knockdown cells were found (Fig. 4B, left and middle). To quantify the effect of Rab27a-knockdown on PMA-induced NET formation, the ratio of cells with individual nuclear stage at 4 h after PMA treatment was calculated (Fig. 4B, right). Most of Rab27a-knockdown cells retained lubulated nuclei after PMA treatment and the appearance of Sytox Green-positive cells was completely inhibited. Collectively, these results indicate that Rab27a plays an essential role in PMA-induced NET formation.

To identify the mechanism by which Rab27a promotes NET formation, ROS production after PMA treatment was quantified using flow cytometry. As a time course study indicated that the maximal fluorescence intensity was obtained around 20 to 30 min (data not shown) after the treatment, we compared the fluorescence intensity between control and Rab27a-knockdown cells during that time period. Rab27a-knockdown significantly suppressed PMA-induced hypochlorite production (Fig. 4C, left). General ROS production was further analyzed using CM-H_2_DCFDA, and reduced ROS production was confirmed in Rab27a-knockdown cells (Fig. 4C, right). These data suggest that Rab27a participates in NET formation via promoting ROS production and the activation process of NADPH oxidase.

### Rab27a is Important in *C. albicans*-induced NET Formation Mediated by NADPH Oxidase Activation

**Figure 5:**
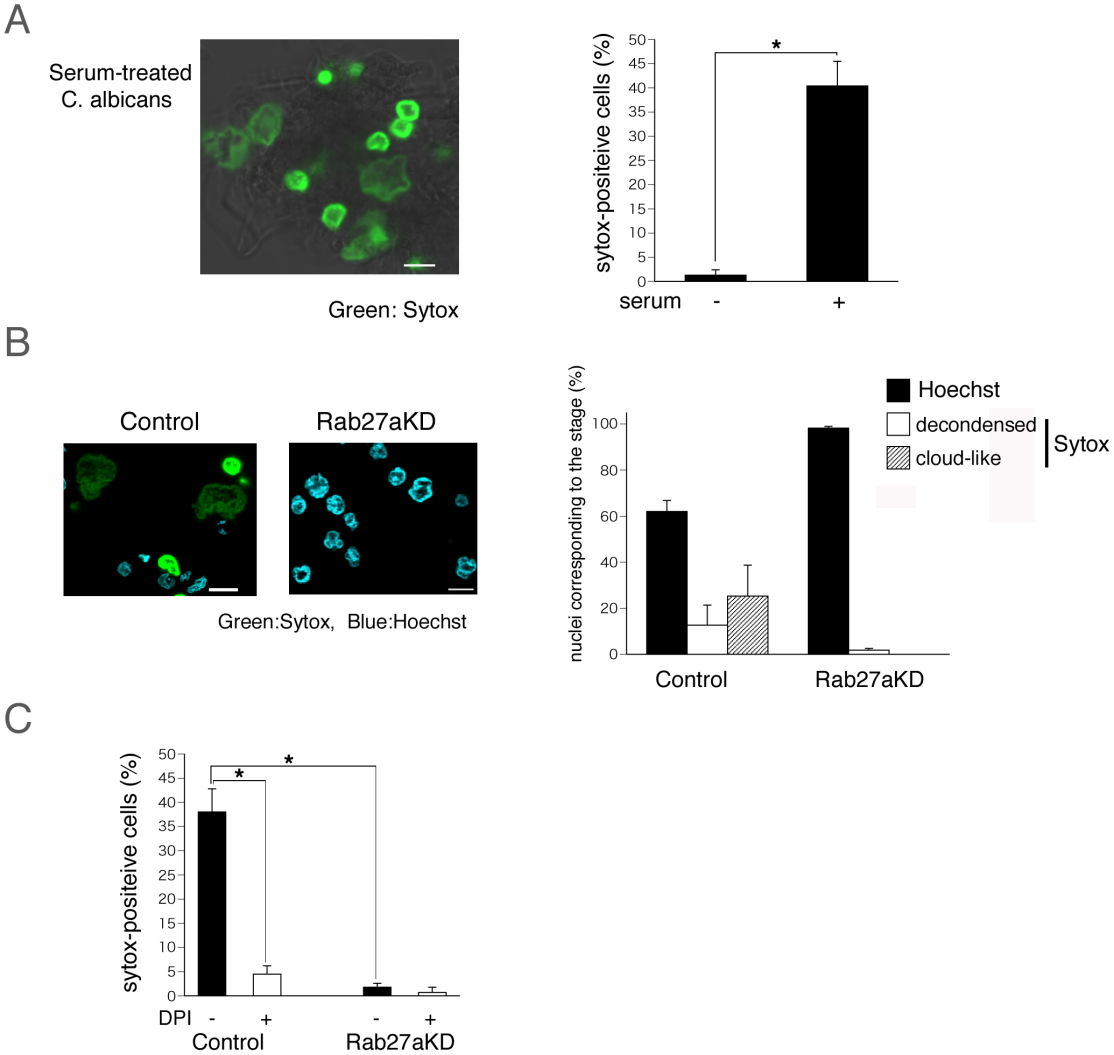
NET formation induced by complement-mediated phagocytosis against the invasion of *C. albicans*. (A) Serum-treated or non-treated *C. albicans* were added to neutrophil-like differentiated HL60 cells (the ratio of *C. albicans* to cell; 5∶1) in the presence of Sytox Green for 3 h at 37°C. Left panel shows representative fluorescence images and right panel shows frequency of Sytox green-positeve cells. Green shows Sytox Green staining. (B) Serum-treated *C. albicans* were added to neutrophil-like differentiated control cells and Rab27a-knockdown cells in the presence of both Hoechst 33342 and Sytox Green for 3 h (The ratio of *C. albicans* to cell; 5∶1). Left panel shows representative fluorescence images. Blue shows Hoechst 33342 staining and Green Sytox Green. Right panel shows the percentage of the cells in the individual stage (black: Hoechst 33342–positive; white: Sytox Green-positive and decondensed; shaded area: Sytox Green-positive and cloud-like shape). (C) Serum-treated *C. albicans* were added to neutrophil-like differentiated control cells and Rab27a-knockdown cells in the presence or absence of DPI for 3 h. Frequency of Sytox green-positive cells are shown. In (A) and (B), Scale bars indicate 10 µm. In (A), (B) and (C), the results of Rab27aKD clone 1 and Control clone 1 are shown and the data are the mean with SD from three independent experiments. Asterisks (*) mean that the difference is statistically significant (p values <0.01).

Next, we attempted to elucidate the process of NET formation induced by infection with the pathogenic fungus *C. albicans*. Serum-treated or untreated *C. albicans* were added to cultures of neutrophil-like differentiated HL60 cells (5∶1 ratio of *C. albicans* to HL60 cells) and incubated for 3 h at 37°C. As shown in Figure 5A, in the presence of serum-treated *C. albicans*, about 40% of the cells were Sytox Green-positive, and most of these cells further converted to cloud-like structures. In contrast, untreated *C. albicans* did not induce NET formation, suggesting that complement-mediated phagocytosis accelerates NET formation. During 3 h of culture in the presence of serum-treated *C. albicans*, one-third of the control cells became Sytox Green-positive, but Rab27a-knockdown cells did not (Fig. 5B), indicating that Rab27a is required for NET formation triggered by serum-treated *C. albicans*. We also tested whether *C. albicans-*induced NET formation is sensitive to the NADPH oxidase inhibitor DPI. As shown in Figure 5C, pretreatment with DPI markedly reduced the proportion of Sytox Green-positive cells during coculture with *C. albicans*, suggesting that ROS production via NADPH oxidase is essential for serum-treated *C. albicans*-induced NET formation, and that Rab27a plays a critical role in the ROS delivery system.

## Discussion

In this study, we found that Rab27a plays critical roles in both NET formation and complement-mediated phagocytosis via ROS production. As shown in Figure 2A, Rab27a is not involved in the initial step of complement-mediated phagocytosis in neutrophil-like differentiated HL60 cells, in contrast to macrophage-like differentiated cells [6], probably because the machinery of neutrophil phagocytosis, including the formation of phagosome, is different from that of macrophages [28].

We also examined the role of Rab27a after the process of phagosome formation. As shown in Figures 2B and 2C, Rab27a-knockdown clearly suppressed the increase in the number of hypochlorite (MPO product)-positive phagosomes, indicating that Rab27a is required for MPO activation after phagosome formation. One possible mechanism is raised that recruitment of NADPH oxidase to phagosomes is dependent on Rab27a. Jancic et al. demonstrated using Rab27a-deficient ashen mice that in dendritic cells, Rab27a-dependent recruitment of NADPH oxidase prevents acidification of the phagosome and limits proteolytic activity for antigen cross-presentation [7]. Similar mechanisms may also occur in neutrophils. As another possible mechanism, Rab27a might directly contribute to the activation process of NADPH oxidase. The role of Rab27a in NADPH oxidse activation and ROS production at the plasma membrane was previously reported in Rab27a-deficient murine neutrophils [29]. In the present study, we additionally demonstrate a novel role of Rab27a in ROS production at the phagosomes in complement receptor-mediated phagocytosis.

The effect of Rab27a on ROS production during phagocytosis suggests that Rab27a contributes to NET formation. NETs are extracellular structures composed of chromatin and granule proteins that bind and kill microorganisms [9]–[11]. Lots of physiological inducers of NET formation have been reported including infection with bacteria, fungi, parasites, and HIV [30]–[33], as well as diverse ligands that stimulate neutrophil receptors, such as cytokine and chemokine receptors [34], [35]. Previous studies on the signaling pathways of NET formation indicate a complex process that differs depending on the stimulation, whereas the importance of ROS production has been established in various NET formation systems. [15]–[17]. PMA is a compound used most frequently to induce NET formation. PMA activates PKC family enzymes, and PKC is directly responsible for activation of NADPH oxidase [17].

First, to estimate whether HL60 acts as a surrogate neutrophils in NET formation process, we treated both primary human neutrophils and neutrophil-like differentiated HL60 cells with PMA, and 1) the amount of extracellularly released histone H3 [24] and 2) chromatin decondensation process were compared between both cells. As shown in Figure 3A, time-dependent release of histone H3 occurred in neutrophil-like differentiated HL60 cells as well as primary human neutrophils. Furthermore, live cell imaging exhibited a similar chromatin decondensation process in both cells. From these results, we judged that neutrophil-like differentiated HL60 cells potentially act as a model of surrogate neutrophils in NET formation process as previously reported [24], [25].

To know the effects of Rab27a on NET formation process, citrullination of the arginine in position 3 of histone H4, a sign of chromatin decondensation was assesed by immunoblotting and microscopic analysis. PMA-dependent increase of H4cit3 was hardly seen in Rab27a-knockdown cells (Fig. 4A). Next, the effect of Rab27a-knockdown on chromatin decondensation process was analyzed. As shown in Figure 3, PMA stimulation sequentially led to decondensation of chromatin into NET-like structure in neutrophil-like differentiated HL60 cells. Four hours after PMA stimulation most of Rab27a-knockdown cells revealed lobulated nuclei stained with cell-permeable dye but about a half of the control cells showed decondensed nuclei (Fig. 4B). These results indicate that Rab27a-knockdown inhibits PMA-induced NET formation in the early stage. To clarify the mechanisms by which Rab27a contributes to the early phase of NET formation, we focused on PMA-induced ROS production. Both hypochlorite production and general ROS production were significantly suppressed after PMA stimulation in Rab27a-knockdown cells (Fig. 4C). These results suggest that Rab27a plays a critical role in the upregulation of ROS production during the early stage of PMA-induced NET formation. In contrast, Catz et al. insisted that Rab27a is not required for NET formation from the data using Rab27a-deficient murine neutrophils [36]. One possible explanation for the conflicted data is the difference of species used in the study. Akong-Moore et al. found that pharmacologic inhibition of MPO decreased NETosis in human neutrophils but not in murine neutrophils and they raised an example of species-specific differences in phenotypes of NETs. Together with our data, it is important to recognize that the regulatory contributions of MPO and HOCl or contribution of Rab27a may be different between murine and human neutrophils, although the mouse model has proved a valuable tool for exploring the biology of NETs in infection and inflammation models [37].

Furthermore, a variety of signaling molecules act at downstream of Rab27a and lead to diverse neutrophil functions [38]. It is suggested that Rab27a participates in a direct activation process of NADPH oxidase such as complex formation which consists of 5 phox units containing NOX2 and Rac. Slp1/JFC1, an effector protein of Rab27a, discovered as a tandem C2 domain-containing protein associated with the leukocyte NADPH oxidase may act to proceed NADPH oxidase activation by carrying p67phox to the membrane at downstream of Rab27a [39]. In contrast, as for another Rab27a effector molecule Munc13-4, NET formation is enhanced in its deficient neutrophils [40]. The change of balance between the downstream effectors of Rab27a may determine the function of neutrophils including NET formation. In additon, there may be raised the possibility that Rab27a regulates membrane fusion between NADPH oxidase-containing granules and other granules, which leads to activation of NADPH oxidase.

In addition to the indispensable requirement of NADPH oxidase, it is reported that upon activaiton and ROS production, neutrophil elastase and MPO stored in the azurophilic granules translocate to the nucleus during NET formation [18], [19].

Finally, we attempted to determine whether Rab27a contributes to NET formation triggered by *C. albicans* infection (Fig. 5). As we confirmed that complement-mediated phagocytosis accelerates NET formation, we used serum-treated *C. albicans* for this portion of the study (Fig. 5A). Our results showed that Rab27a is required for NET formation triggered by serum-treated *C. albicans*, and that ROS production via NADPH oxidase is essential for this process (Figs. 5B, 5C). Rab27a thus plays a critical role in *C. albicans* infection-induced NET formation through mediation of the ROS delivery system.

In conclusion, we demonstrate that Rab27a regulates the function of NADPH oxidase and subsequent ROS production, and is thus essential for both PMA- and *C. albicans*-induced NET formation.
